# A network perspective on patient experiences and health status: the Medical Expenditure Panel Survey 2004 to 2011

**DOI:** 10.1186/s12913-017-2496-5

**Published:** 2017-08-22

**Authors:** Yi-Sheng Chao, Hau-tieng Wu, Marco Scutari, Tai-Shen Chen, Chao-Jung Wu, Madeleine Durand, Antoine Boivin

**Affiliations:** 1 Centre de recherche du centre hospitalier de l’Université de Montréal (CRCHUM), Université de Montréal, Montreal, Canada; 2 Department of Mathematics, University of Toronto, Toronto, Canada; 3 Department of Statistics, University of Oxford, Oxford, UK; 4 Graduate School of Agricultural and Life Sciences, University of Tokyo, Tokyo, Japan; 5 Université du Québec à Montréal, Montreal, Canada; 6 Département de médecine de famille, Centre de recherche du centre hospitalier de l’Université de Montréal (CRCHUM), Institut de recherche en santé publique (IRSPUM), Université de Montréal, Montreal, Canada

**Keywords:** Bayesian network, Patient experience, Health status

## Abstract

**Background:**

There is a growing emphasis on the need to engage patients in order to improve the quality of health care and improve health outcomes. However, we are still lacking a comprehensive understanding on how different measures of patient experiences interact with one another or relate to health status. This study takes a network perspective to 1) study the associations between patient characteristics and patient experience in health care and 2) identify factors that could be prioritized to improve health status.

**Methods:**

This study uses data from the two-year panels from the Medical Expenditure Panel Survey (MEPS) initiated between 2004 and 2011 in the United States. The 88 variables regarding patient health and experience with health care were identified through the MEPS documentation. Sex, age, race/ethnicity, and years of education were also included for analysis. The *bnlearn* package within R (v3.20) was used to 1) identify the structure of the network of variables, 2) assess the model fit of candidate algorithms, 3) cross-validate the network, and 4) fit conditional probabilities with the given structure.

**Results:**

There were 51,023 MEPS interviewees aged 18 to 85 years (mean = 44, 95% CI = 43.9 to 44.2), with years of education ranging from 1 to 19 (mean = 7.4, 95% CI = 7.40 to 7.46). Among all, 55% and 74% were female and white, respectively. There were nine networks identified and 17 variables not linked to others, including death in the second years, sex, entry years to the MEPS, and relations of proxies. The health status in the second years was directly linked to that in the first years. The health care ratings were associated with how often professionals listened to them and whether professionals’ explanation was understandable.

**Conclusions:**

It is feasible to construct Bayesian networks with information on patient characteristics and experiences in health care. Network models help to identify significant predictors of health care quality ratings. With temporal relationships established, the structure of the variables can be meaningful for health policy researchers, who search for one or a few key priorities to initiate interventions or health care quality improvement programs.

**Electronic supplementary material:**

The online version of this article (doi:10.1186/s12913-017-2496-5) contains supplementary material, which is available to authorized users.

## Background

Engaging patients is an important element of healthcare, as it improves patient experience and thus leads to improved health outcomes [1]. For example, experiences in the timeliness and perceived quality of health care and communication with physicians are measured with the Consumer Assessment of Health Plans Study (CAHPS) questionnaire [2]. The perceived quality of primary care has been linked to outcomes, such as emergency room visits [3] and other health care use [4]. The recent focus on patient experience and patient-oriented practice, especially engagement, aims to improve health care quality and patient health [5, 6]. However, there are several problems in studying the relationship between patient experience and health outcomes. One of the issue is that patient experiences may be simplified into a single dimension [7]. This may oversimplify the complexity of patient experiences in health care and overlook the opportunities that we can take advantage of to improve healthcare quality and patient health [7]. On the contrary, there is no common measure or universally agreed - upon definition of patient engagement [8] or patient satisfaction, each an important aspect of patient experience [7]. In fact, there are various experience measures and definitions proposed to identify important components or priorities that we can adapt to improve patient centeredness or engage patient effectively [8]. Only a few of them show promising results in validity or reliability, including the CAHPS [9]. Furthermore, the input of all types of health professionals may not be properly measured in questionnaires. For example, the communication with providers other than doctors may not be considered while assessing patient experience [7]. This can underestimate the input and effectiveness of patient-provider communication to improve patient health [7].

Occasionally, the objective of improving patient experience and subsequently health outcomes may be in conflict [7]. One reason is that the priorities identified via various methods may not be compatible with each other [10]. There isn’t enough evidence to help us understand whether there are pathways or networks to link individual characteristics or experiences to better outcomes in health care. Whether multiplicative benefits through changes in upstream policies or interventions can be achieved remains unclear [11, 12]. This problem is aggravated by the fact that there are few studies with sufficient samples to systematically identify the key factors for patient experience improvement [8].

To address these problems, we adopt a network approach to examine all possible inter-dependence of these measures of patient experience and health outcomes in a large population, while also taking into account individual characteristics. This can help us to identify potential intervention priorities as well as avoid incurring undesirable or adverse interactions between them. This study aims to 1) construct a Bayesian network model with individual characteristics and commonly used measures of patient experience, especially quality of care in the CAHPS, and 2) illustrate the relationships between patient experience and health outcomes through graphics and probability distributions.

## Methods

This study uses data from the Medical Expenditure Panel Survey (MEPS) that was implemented with a focus on both self-perceived health status and patient experience with health care [13]. The MEPS staff interviewed non-institutionalized civilians in the United States since 1996 [14]. The MEPS provided a nationally representative sample with an oversample of blacks and Hispanics [14]. The interviewees were followed up for 2 years during each MEPS panels [14].

**Fig. 1:**
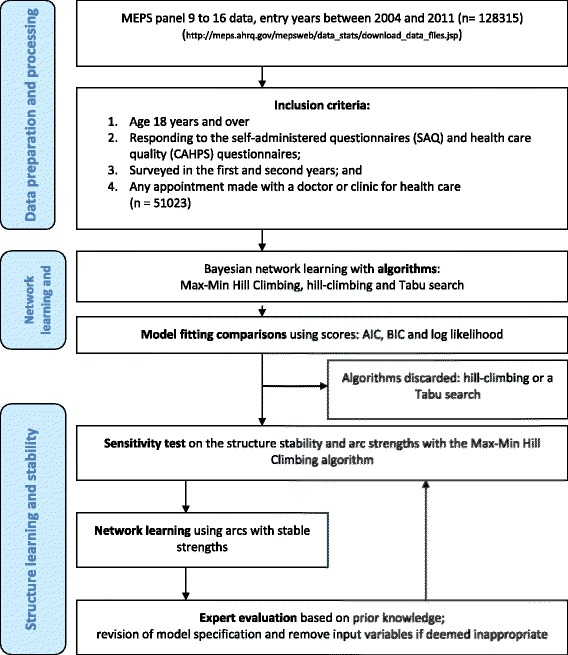
The flowchart of learning the network structure with variables regarding health care quality and patient-reported outcomes from the Medical Expenditure Panel Survey (MEPS) 2004 to 2011

The questions about patient-perceived health and experience in health care were asked once per year or twice in the two-year panels [14]. The questionnaires were modified and new variables were added over time. To ensure the consistency of the variables in the MEPS panels, only data from two-year panels initiated between 2004 and 2011 were used. Because of the lack of adequate tools to adjust for complex survey design under graphic models, all statistics in this study were unweighted and not nationally representative. The flowchart of data processing and analysis is in Fig. 1.

### Inclusion and exclusion criteria

This study included those age 18 years and over. Only those who made an appointment with a doctor or clinic for health care were included. Those with missing data in the following section were not included: the self-administered questionnaire (SAQ) that contained questions on patient experience of care (Consumer Assessment of Healthcare Providers and Systems, CAHPS) and three types of self-reported physical or mental health status: 1) Short-Form 12 Version 2, SF-12v2 along with the Physical Component Summary (PCS) and the Mental Component Summary (MCS) of the SF-12v2, 2) the Kessler Index (K6) of non-specific psychological distress; and 3) the Patient Health Questionnaire, PHQ-2 [15]. Missing data were defined as the following answer categories: no data in round, refused, don’t know, or not ascertained. If subjects were not eligible for specific questions, their replies were coded as not applicable (see Additional file 1: Appendix 1 for proportions of ineligibility). For example, interviewees were asked whether they had experienced an illness or injury that had required immediate care. This question might not apply to all surveyed individuals.

### Variable inclusion

The list of variables regarding patient experience, especially health care ratings, and patient-reported outcomes were selected and categorized according to the MEPS documentation (see Additional file 1: Appendix 1 for details) [15]. The patient-reported rating of health care ranging from zero to 10 was provided by those who had visited health care professionals (variable name: adhecr2 and adhecr4 for the first and second years respectively). Individual health status, measured by SF-12v2 (adgenh2 and adgenh4 for the first and second years respectively), were reported in five categories: poor, fair, good, very good, and excellent. The labels for variables imported from the MEPS documentation were also listed in Table 1. Moreover, sex, age, race/ethnicity, regions (Northeast, Midwest, South and West regions of the United States) and years of education were also included in the network analysis.

**Table 1:** The definitions variables from the Medical Expenditure Panel Survey (MEPS) between 2004 and 2011

Variables		Labels	Categories
died		Died during MEPS panels	
meps		Entry year to the MEPS	
racex		Race	
agey1x		Age as of 12/31	
regiony1		Census region as of 12/31	
ttlpy1x		Person’s total income	
educyr		Years of education when first entered MEPS	
sex		Sex	
First-year variables	Second-year variables		
adappt2	adappt4	Last 12 months: numbers of visits to medical office for care	CAHPS
adcape2	adcape4	Last 4 weeks: felt calm/peaceful	SF-12v2
adclim2	adclim4	Health limits climbing stairs	SF-12v2
addaya2	addaya4	Health limits in moderate activities	SF-12v2
addown2	addown4	Last 4 weeks: felt downhearted or depressed	SF-12v2
addprs2	addprs4	Last 2 weeks: felt down/depressed/hopeless	Patient Health Questionnaire
addrbp2	addrbp4	Last 2 years: whether physician checked blood pressure	General Health
adefrt2	adefrt4	Last 30 days: how often everything was an effort	Non-specific Psychological Distress
adexpl2	adexpl4	Last 12 months: doctor explained in a way that was understandable	CAHPS
adgenh2	adgenh4	Health in general	SF-12v2
adhecr2	adhecr4	Last 12 months: rating of health care	CAHPS
adhope2	adhope4	Last 30 days: how often felt hopeless	Non-specific Psychological Distress
adilcr2	adilcr4	Last 12 months: illness/injury needing immediate care	CAHPS
adilww2	adilww4	Last 12 months: got care when needed in case of illness/injury	CAHPS
adinsa2	adinsa4	Do not need health insurance	Attitudes about Health
adinsb2	adinsb4	Health insurance not worth cost	Attitudes about Health
adintr2	adintr4	Last 2 weeks: little interest in things	Patient Health Questionnaire
adlist2	adlist4	Last 12 months: doctor listened to you	CAHPS
admals2	admals4	Last 4 weeks: as result of mental problems, accomplished less than you would like	SF-12v2
admwlm2	admwlm4	Last 4 weeks: as result of mental problems, did work or other activities less carefully than usual	SF-12v2
adndcr2	adndcr4	Last 12 months: need any care, test, treatment	CAHPS
adnerv2	adnerv4	Last 30 days: how often felt nervous	Non-specific Psychological Distress
adnrgy2	adnrgy4	Last 4 weeks: had a lot of energy	SF-12v2
adnsmk2	adnsmk4	Last 12 months: doctor advised to quit smoking	General Health
adover2	adover4	Can overcome illness without medical help	Attitudes about Health
adpain2	adpain4	Last 4 weeks: pain limits normal work	SF-12v2
adpals2	adpals4	Last 4 weeks: as result of physical health, accomplished less than would like	SF-12v2
adprtm2	adprtm4	Last 12 months: doctor spent enough time with you	CAHPS
adprx2	adprx4	Relationship of proxy to adult	
adpwlm2	adpwlm4	Last 4 weeks: as result of physical health, limited in kind of work or other activities	SF-12v2
adresp2	adresp4	Last 12 months: doctor showed respect	CAHPS
adrest2	adrest4	Last 30 days: how often felt restless	Non-specific Psychological Distress
adrisk2	adrisk4	More likely to take risks	Attitudes about Health
adrtcr2	adrtcr4	Last 12 months: made appointment for routine medical care	CAHPS
adrtww2	adrtww4	Last 12 months: got medical appointment when wanted	CAHPS
adsad2	adsad4	Last 30 days: how often felt sad	Non-specific Psychological Distress
adsmok2	adsmok4	Currently smoke	General Health
adsoca2	adsoca4	Last 4 weeks: health stopped social activity	SF-12v2
adspec2	adspec4	Last 12 months: needed to see specialist	General Health
adwrth2	adwrth4	Last 30 days: how often felt worthless	Non-specific Psychological Distress
k6sum2	k6sum4	Last 30 days: overall rating of feelings	Kessler Index
mcs2	mcs4	Mental component summary	SF-12v2
pcs2	pcs4	Physical component summary	SF-12v2
phq22	phq24	Last 2 weeks: overall rating of feelings	Patient Health Questionnaire

Note: *CAHPS* consumer assessment of Healthcare Providers and Systems, *SF-12* short-form 12

There were five rounds of data collection in each two-year panel. The SAQ was administered during the second and fourth rounds that were approximately in the middle of the years [15]. Therefore, each outcome or experience measure was numbered with one or two to represent first and second-year information.

### Bayesian network

Bayesian networks consist of nodes and arcs that represented variables of interest and the relationships between them [16]. The relationships between variables were described in conditional dependencies and tested with Chi-square tests or other score-based methods [17]. Given its advantage of visual presentation and network structures that were more appropriate to describe interactions between variables, Bayesian networks were used in medical [18], biological [19], and social [20] research to study the conditional dependencies between random variables.

### Layers of variables

The directions between variables were regulated in layers [21]. The higher-layer variables were allowed to be directed to variables of other layers. However, variables of other layers were not permitted to be directed to higher-layer variables. The highest layer was one, and included the following variables: years of entry to the MEPS panels, races, ages in years, regions, education, and sex. The second-layer variables were income, health status, and experience in health care in the first years of the panels. The third-layer variables were deaths in the second years, and the health status and experience in health care in the second years of the panels (see Additional file 1: Appendix 1). In other words, some of the directions between variables were blacklisted in the network modeling [17].

### Model development process

The network modeling followed a previously published development process to review and revise the process [22]. The initial Bayesian network models were developed after data cleaning and missing data assessment [23]. The following steps were taken to finalize the model. First, data-driven models were built and assessed for adequacy according to expert opinions by all authors. This led to some adjustments in the variables to be specified in different layers. The performance of different algorithms was also compared. Second, the expert panel made decisions about 1) the retaining of essential variables for further model selection, 2) the identification of important links between variables, and 3) the validation of the conditional probability distributions based on prior knowledge on the research of patient experience and engagement. After discussion and model re-specification, the Bayesian network models were rerun to obtain stable network structures based on 10-fold cross-validation.

The temporal relationships between first-year and second-year variables in the MEPS panels were considered in the model development process. In some of the complex time series studies based on network modeling, the relationships among variables of different time points were assumed to be similar. For example, the relationships between insulin adjustment and other related variables were assumed similar across different time points [24]. However, we considered that there was limited evidence to justify imposing similar network structures to the first-year and second-year variables, given this to be one of the first studies using Bayesian network modeling with patient experience data.

### Bayesian network implementation

The *bnlearn* package [25] available within R environment (v3.20 released in April 2015) was used to 1) apply several of the best heuristic algorithms, including Max-Min Hill Climbing that obtained the best scores in network modeling with the MEPS data [21] (see Additional file 2: Appendix 2 for the scores), 2) verify the stabilities and strengths of network arcs through averaging 200 bootstrapped networks, and 3) query the conditional probability distributions in the finalized network, and 4) illustrate the final networks with visualization tools [17]. If the Bayesian network models were found to be inadequate by expert opinions in any step of the development process, these procedures were rerun to obtain finalized network models.

### Correlations between variables and cross-group comparisons

In addition to Bayesian network modeling, the associations between variables were also determined by the correlation coefficients in Spearman’s correlation tests. The differences in continuous and categorical variables across countries or parent variables were also tested with Student’s t and Chi-square tests, respectively. The level of significance was at 0.05 level at two tails.

## Results

The demographic characteristics of the MEPS participants are listed in Table 2. Between 2004 and 2011, there were 51,023 MEPS participants aged 18 to 85 years (mean = 44.1, 95% CI = 43.9 to 44.2). The years of education ranged from 1 to 18 years (7.4, 95% CI = 7.40 to 7.46). The proportion of female participants was 55% and did not change significantly across MEPS panels. The majority of those sampled were white, at 76%, with the largest sample, 38%, being from the South.

**Table 2:** The demographic characteristics of the MEPS interviewees

Year	N	Age (years)	(95% CI)	Education (years)	(95% CI)	Female (%)	Race/ethnicity (%)						Regions (%)			
							White	Black	Asian	American Indians/Alaska natives	Native Hawaiian/Pacific islanders	Multiple races	Northeast	Midwest	South	West
2004	6414	43.80	(43.40 to 44.20)	7.41	(7.32 to 7.50)	54.58%	81.03%	11.83%	0.86%	4.23%	0.44%	1.62%	14.76%	21.02%	39.87%	24.35%
2005	6246	44.51	(44.10 to 44.92)	7.47	(7.39 to 7.56)	54.00%	79.35%	14.35%	0.78%	3.86%	0.26%	1.41%	14.49%	21.81%	36.95%	26.75%
2006	6182	44.49	(44.08 to 44.90)	7.48	(7.40 to 7.57)	53.87%	79.25%	13.52%	0.74%	4.63%	0.37%	1.49%	14.17%	22.45%	37.53%	25.85%
2007	4542	44.11	(43.63 to 44.59)	7.52	(7.42 to 7.61)	52.77%	78.49%	12.53%	0.59%	6.36%	0.37%	1.65%	15.24%	21.07%	37.98%	25.72%
2008	7231	42.91	(42.53 to 43.28)	7.45	(7.37 to 7.53)	53.93%	72.67%	17.78%	1.02%	6.60%	0.25%	1.67%	14.58%	20.20%	38.45%	26.77%
2009	6597	44.59	(44.19 to 45.00)	7.41	(7.33 to 7.49)	52.87%	73.44%	16.77%	0.97%	7.49%	0.24%	1.09%	14.22%	21.56%	38.08%	26.15%
2010	5979	44.23	(43.81 to 44.66)	7.38	(7.30 to 7.46)	53.05%	71.57%	17.41%	0.92%	7.88%	0.74%	1.49%	15.52%	21.34%	37.18%	25.96%
2011	7832	44.12	(43.75 to 44.50)	7.38	(7.31 to 7.45)	53.20%	72.50%	17.71%	0.00%	7.12%	0.00%	2.67%	15.12%	21.02%	37.12%	26.75%
All	51,023	44.07	(43.93 to 44.22)	7.43	(7.40 to 7.46)	53.56%	75.80%	15.44%	0.73%	6.05%	0.32%	1.67%	14.75%	21.28%	37.89%	26.08%

Note: MEPS years = the years of entry to the Medical Expenditure Panel Survey. N = sample size

### Network overview

**Fig. 2:**
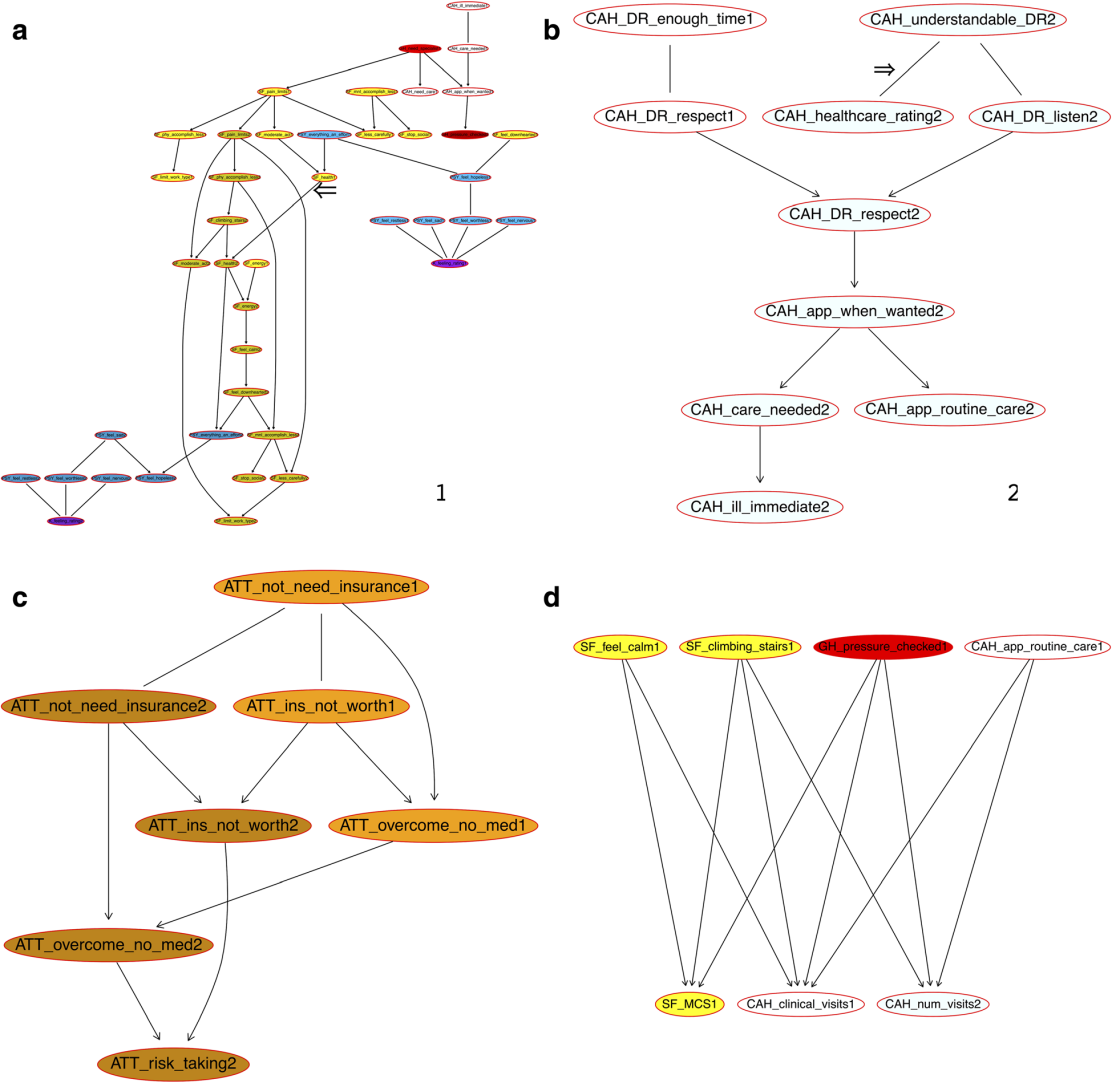
The networks of patient-reported outcomes and health care quality indicators among the MEPS interviewees age 18 years and over between 2004 and 2011. **a** The largest network consisting of 42 variables. **b** The second largest network consisting of only the CAHPS variables about the patient experiences in health care. **c** The network consisting of the variables related to attitudes about health and insurance. **d** The network consisting of the variables related to health care needs and appointment making. Note: The Bayesian network learned with the Max-Min Hill Climbing (MMHC) algorithm and 10-fold cross-validation. *Transparent* node color = health care quality (CAHPS) and demographic variables; *red* = general health; *yellow* = SF12; *blue* = non-specific psychological stress; *pale green* = patient health questionnaire (PHQ-2); *orange* = attitude about health; and *purple* = Kessler Index. The numbers in the end of the short names of the variables are the years of the Medical Expenditure Panel Survey that follows up individuals for two years. The shaded colors are for the year-2 variables only

There were nine networks identified and 17 variables were not linked to any others, including death in the second years, sex, entry years to the MEPS, and relations of proxies (see Additional file 1: Appendix 1 for details; see Additional file 3: Appendix 3, Additional file 4: Appendix 4 and Additional file 5: Appendix 5 for all networks). Variables of different categories tended to group in various networks. The largest network contained 42 variables, of which 22 were the health status measured by the SF12v2 and 12 measures of non-specific psychological distress (Figure 2a). The second largest network consisted of ten CAHPS variables (Figure 2b). The third and fourth largest networks each had seven variables: one was related to the interactions between different types of attitudes toward health and the other was related to health care needs and appointment making (Fig. 2c and d respectively). The other networks contained three variables or less (see Additional file 3: Appendix 3, Additional file 4: Appendix 4 and Additional file 5: Appendix 5 for all networks).

### Patient experience: rating of healthcare

The healthcare ratings in the first years of the MEPS panels was directly linked to whether health professionals listened to patients (marked by an arrow, p 6 in Additional file 3: Appendix 3). Healthcare was rated higher when professionals listened to the patients more frequently. The same figure indicated that patients found professionals more understandable when the professionals listened to them more frequently.

**Fig. 3:**
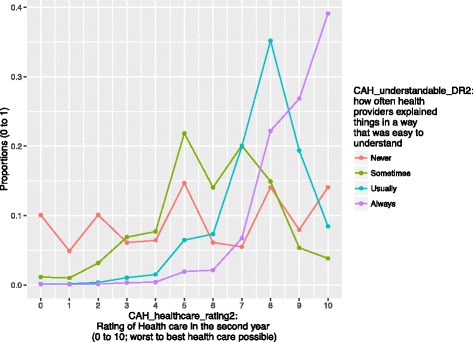
The patient-reported ratings of the health care quality in the second years. Note: Chi-square = 69,556, *p* < 0.001

In contrast, the patient-reported healthcare ratings in the second years was linked to whether the health professionals explained their conditions in a way that they understood (the arc marked by an arrow, Fig. 2b and p 2 in Additional file 3: Appendix 3). The probability distributions of the healthcare ratings were shown in Fig. 3. The more frequently the health providers explained things in a way that was easy to understand, the more likely the patients were to rate health care higher.

### Health status

**Fig. 4:**
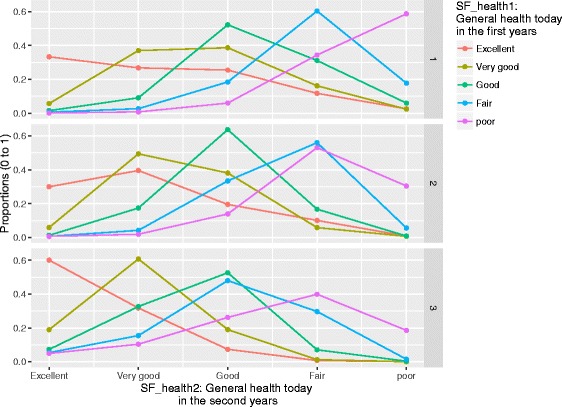
The probability distributions of the general health status in the second years of panels. Note: general health today in the first years of the MEPS panels was significantly associated with general health today in the second years (*p* < 0.001 for both). The numbers on the right side of each chart representing the degree of health limiting climbing stairs according to the SF12v2 questionnaire: 1 = limited a lot, 2 = limited a little, and 3 = not limited

The health status in the first and second years was directly linked to one another in Fig. 2a, the arc marked with a arrow. The probability distributions of general health status in the first and second years were shown in Fig. 4. More than 47% of the individuals maintained the same categories of health status throughout the two-year panels. There were two variables linked to health status in the first years: how often individuals felt everything was an effort and whether health status limited moderate activities. How often individuals felt everything an effort is a question to assess non-specific psychological stress. If patients felt everything an effort more often or more limitation on moderate activities due to health, they were more likely to report a worse health status in the first year. The probability distribution of the health status in the second years was related to health status in the first year and whether their health limited climbing stairs in the second years.

### Connection between first-year and second-year variables

There were limited connections between the first-year and second-year variables. In addition to the link between health status in the first and second years, there were ten other arcs linking first-year and second-year variables. First, if individuals had a lot of energy for a majority of the time during the first year, they tended to feel the same in the second year. Second, if patients were able to make medical appointments when desired in the first year, they were more likely to report having blood pressure checked by health professionals in the second year. Third, the degree to which pain limited normal work in the first year was related to the same variable in the second years. Fourth, the more frequently health professionals showed respect to patients in the first year, the more likely patients were to report more respect to them in the second years. Another three variables were related to the association with the number of visits to medical officers for care in the second years. The last three were the linkages between attitudes about health. Patients’ attitudes about whether they needed insurance, whether insurance was worth costs, and whether they could overcome illness without medical help were consistent in the first and second years.

## Discussion

This study shows that network modeling is both feasible and useful for further policy or academic research. The measures of patient experience and physical or mental health are interconnected across time. The results not only show the complexity in patients’ interactions with healthcare systems, but also point to possible approaches to navigating the intricacies of these interactions. The first important finding is that measures of patient experiences and health status are interconnected, but only to a certain degree. The largest network consists of 42 variables that are predominantly dimensions of mental and physical health measured by SF12v2, non-specific psychological distress, and Kessler scale, along with three measures of patient experience and two general health questions. In this network, self-rated health status in the first and second years is linked. The temporal associations of health status across different time points is verified by previous studies [26].

Second, 13 patient experience variables measured by the CAHPS are included in two separate networks, while seven others are included in two other networks that include measures of health status and SF12v2 functional status. The association between health care rating and understandable explanation by providers [7] can be found in the first or second years. The networks in this study show that age, education or income may not have extensive connection with patient experience in health care, if conditional dependencies taken into account. This is different from previous studies that use regression models and show the associations between patient experience and individual characteristics especially age and sex [7, 27]. Other researchers find that the degree to which patients engage in their care could be explained predominantly by income, with race/ethnicity playing a lesser role [28]. The differences in the results from various methods are expected and the network perspective shows that the inter-dependencies between patient experience measures may need to be considered in regression models as well.

Third, there seem to be key arcs that link health status and patient engagement across time. The first- and second-year variables of health status, how often providers show respect to what patients have to say, whether pain limits normal work, and attitudes about insurance in the first and second year, are connected. The other measures are not well connected across time. 

In addition, this study highlights some of the undervalued associations and opportunities to improve both patient experience and health status. For example, there are extensive interactions between functional status and psychological stress. The importance of mental status has been well demonstrated, [29] and the findings suggest that some measures of the psychological stress may be more important than others. For example, feeling everything is an effort is directly linked to self-reported health status.

### Strengths and limitations

This network approach is useful to handle a large number of variables with or without prior knowledge in the interactions or interdependencies between them [30]. The visual presentation is appealing for the audience, who are interested in exploring interactions between variables or measures of patient experience.

Despite the large sample size and standardized questionnaires used in the MEPS, there are several limitations in the newly identified networks. First, the MEPS is designed to produce nationally representative statistics for the civilians in the United States through the adjustment of the survey design [14]. However, it is not feasible to account for the survey design in the Bayesian network models [17] and certain population groups may be overrepresented. This can limit the generalizability of the results. Second, the research tools, Bayesian networks and graphic models, may not be widely known to health policymakers or researchers, who are familiar with regression models that summarize the significance and associations of all predictors towards a single outcome.

Third, one inherent difficulty in health care quality research with observational data is that potential interventions were not randomly assigned and healthcare is rated only by those with any exposure to health care systems [15]. It is unclear how the causes of health care consumption, chronic or acute, will relate or influence the ratings of health care, whether through self-selection, lack of insurance coverage, [31] or other mechanisms. Fourth, the purpose of the MEPS is to understand the health status of populations on a yearly base. The measurement of patient experiences of the last 1 year may be subject to recall bias. The CAHPS questionnaire may be widely used, but remains unspecific to events, such as specialty visits or hospitalization [7]. Fifth, there was no information about whether patients switched health providers that might be important to patients’ experience in health care. Lastly, causal relationships cannot be established with cross-sectional data [17]. This is to be studied and analyzed with trials or interventions in the future.

### Research implications

These findings are important for the planning of future research. First, the identified networks are meaningful for health policy researchers, who search for one or a few priorities to design and initiate interventions on patient experience in health care in order to improve health status and health care quality. For example, there are several variables linked to more than three measures of patient experience and could serve as intervention priorities, such as making doctors’ explanations understandable or improving appointment-making procedures for routine care.

Second, the network also contrasts two distinctive approaches to improve patient experience and health status. The first one is to use immediate parent variables in the network as the targets of intervention. The other is the trickle-down approach [32] that may focus more on the upstream factors that may not have immediate influence on patient experience or health. Instead, it may be of interest for policy makers who aim to improve the overall well-being extensively, through one of the beginning variables in the network, such as whether health professionals spend enough time with patients.

## Conclusion

Bayesian network modeling is feasible with health experience data. A network perspective highlights the interactions between the measures of patient experience in health care. This can be used to identify potential priorities for interventions that aim to improve health status or experience in health care. Researchers evaluate potential interventions by using this model to identify immediate parent variables or distinct upstream-factors. The effectiveness of these two dissimilar concepts will require further testing.

## Additional files

Additional file 1: Appendix 1. Characteristics of the included variables from the Medical Expenditure Panel Survey. The characteristics of the variables included for analysis. (XLSX 14 kb)

Additional file 2: Appendix 2. Scores of the Bayesian network algorithms. The scores of the Bayesian network algorithms, used to assess model fit and select the algorithm for analysis. (XLSX 7 kb)

Additional file 3: Appendix 3. Networks of the measures of patient experiences and health status with the short names of the Medical Expenditure Panel Survey variables in the nodes. The results of the Bayesian network modeling with all connected networks. The short names of the variables are labelled in the nodes. Corresponding variable names can be found in Additional file 1: Appendix 1. (TIFF 36363 kb)

Additional file 4: Appendix 4. Networks of the measures of patient experiences and health status with the short names of the Medical Expenditure Panel Survey variables in the nodes. The results of the Bayesian network modeling with all connected networks. The original variables are labelled in the nodes. Corresponding variable names can be found in Additional file 1: Appendix 1. (PDF 72 kb)

Additional file 5: Appendix 5. Networks of the measures of patient experiences and health status with the short names of the Medical Expenditure Panel Survey variables in the nodes. The results of the Bayesian network modeling with all connected networks. The names of the variables are labelled in the nodes. Corresponding variable names can be found in Additional file 1: Appendix 1. (PDF 76 kb)

## Abbreviations

CAHPS: Consumer Assessment of Healthcare Providers and Systems
K6: Kessler index
MCS: Mental Component Summary
MEPS: Medical Expenditure Panel Survey
PCS: Physical Component Summary
PHQ-2: Patient health questionnaire
SAQ: Self-administered questionnaire
SF12v2: Short-form 12 version 2
