# Supplementary figures and images for: A network perspective on patient experiences and health status: the Medical Expenditure Panel Survey 2004 to 2011
Source: BMC Health Serv Res. 2017 Aug 22;17:579. doi: 10.1186/s12913-017-2496-5 (PMC5567925; doi:10.1186/s12913-017-2496-5)

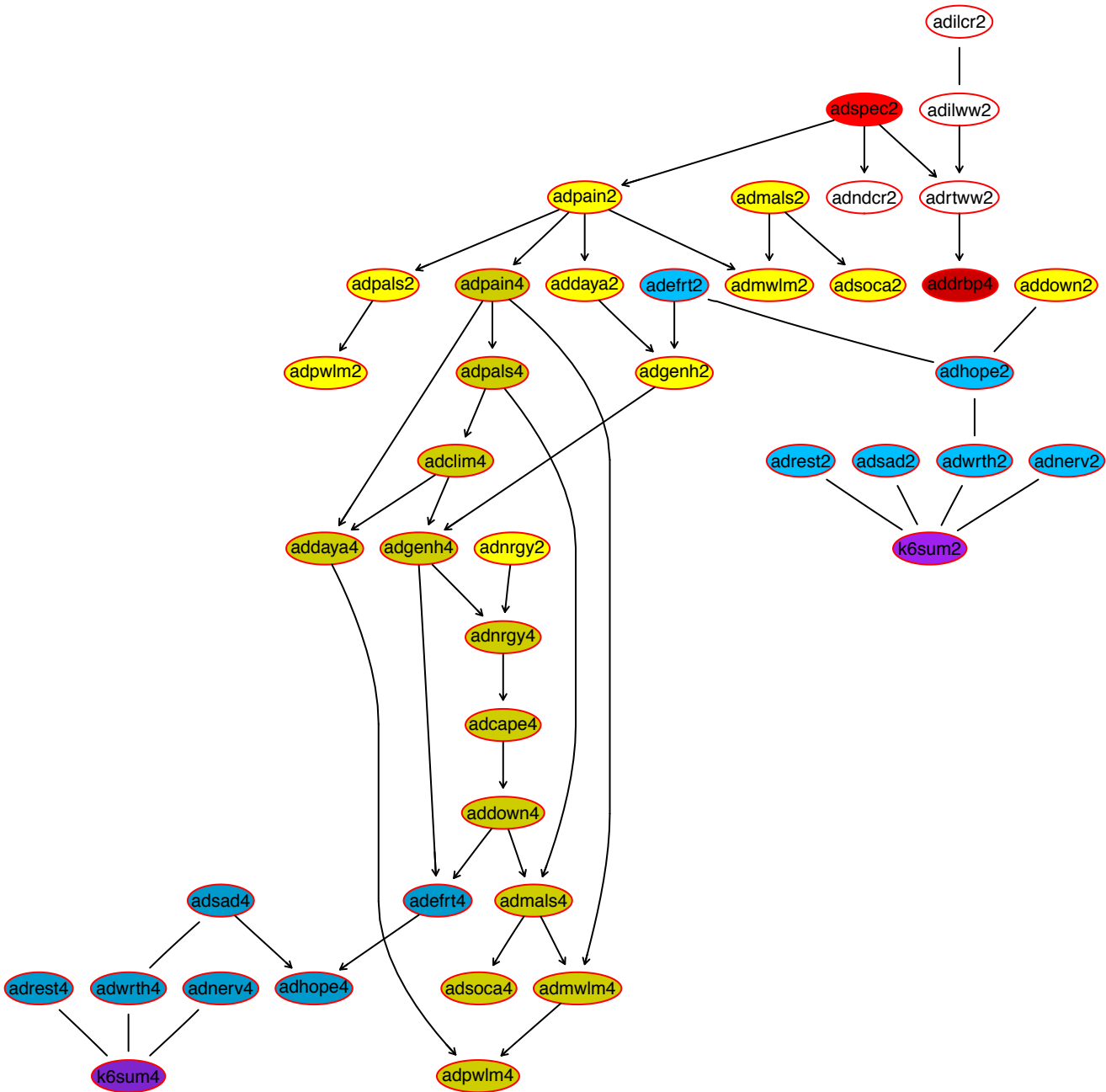

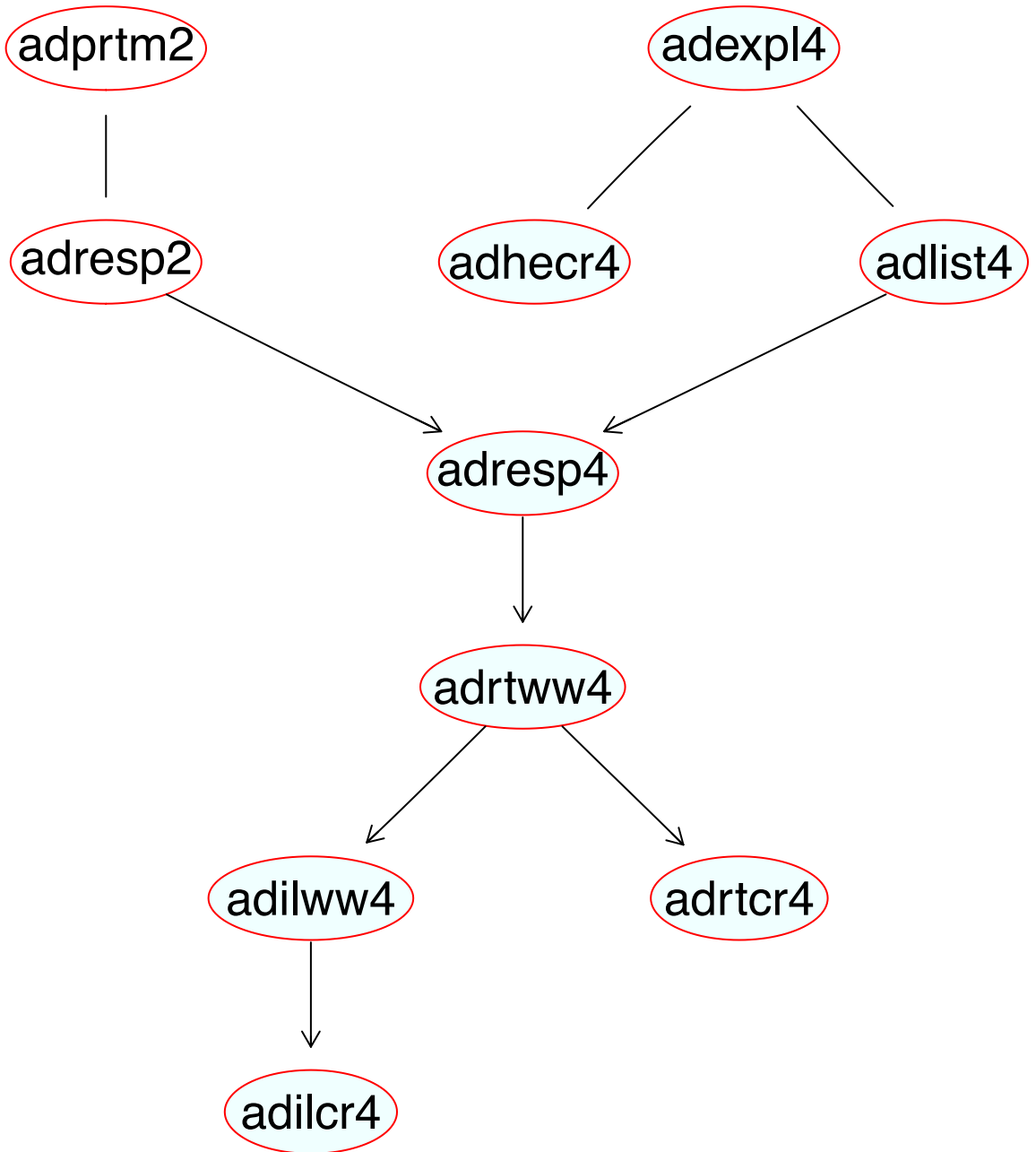

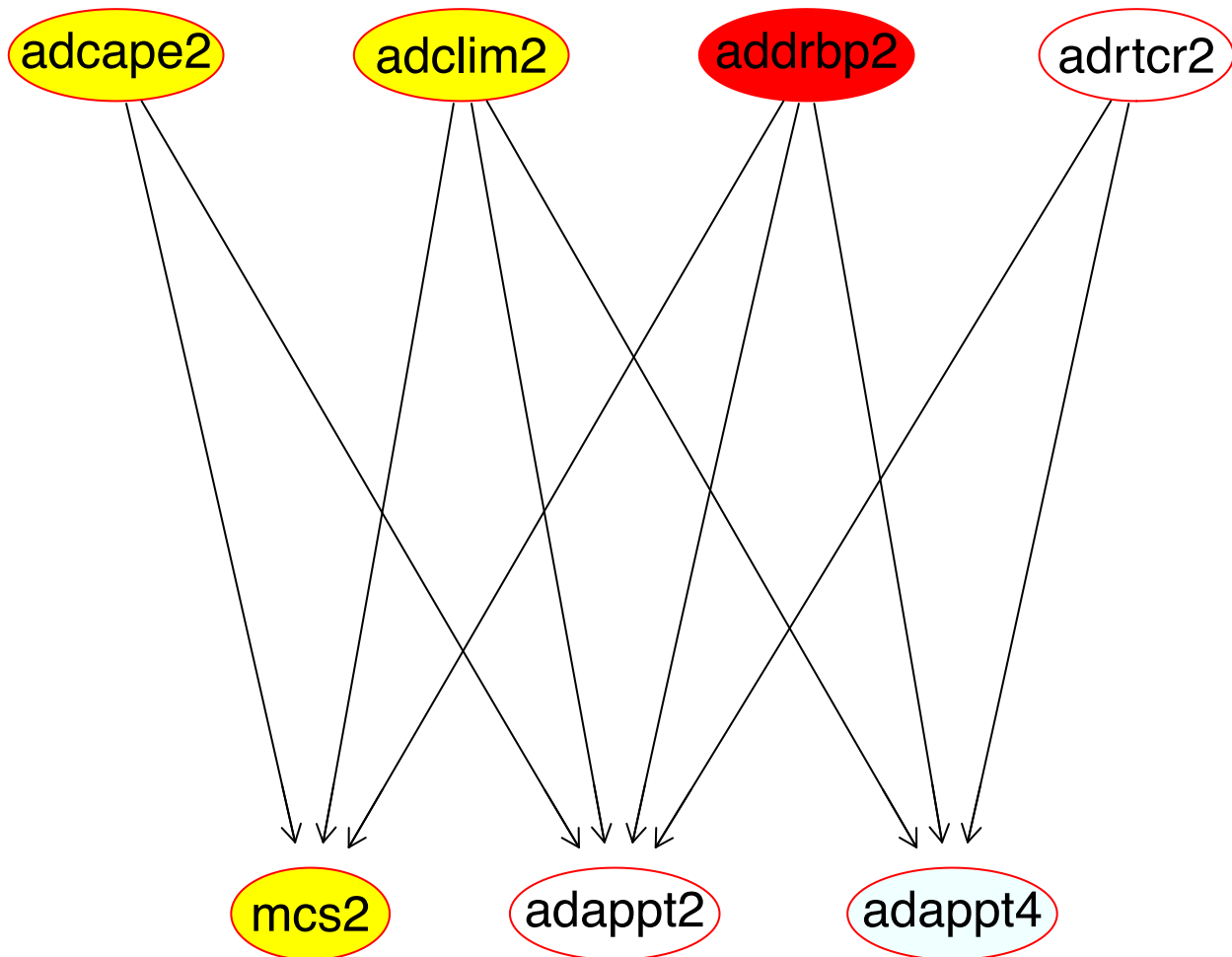

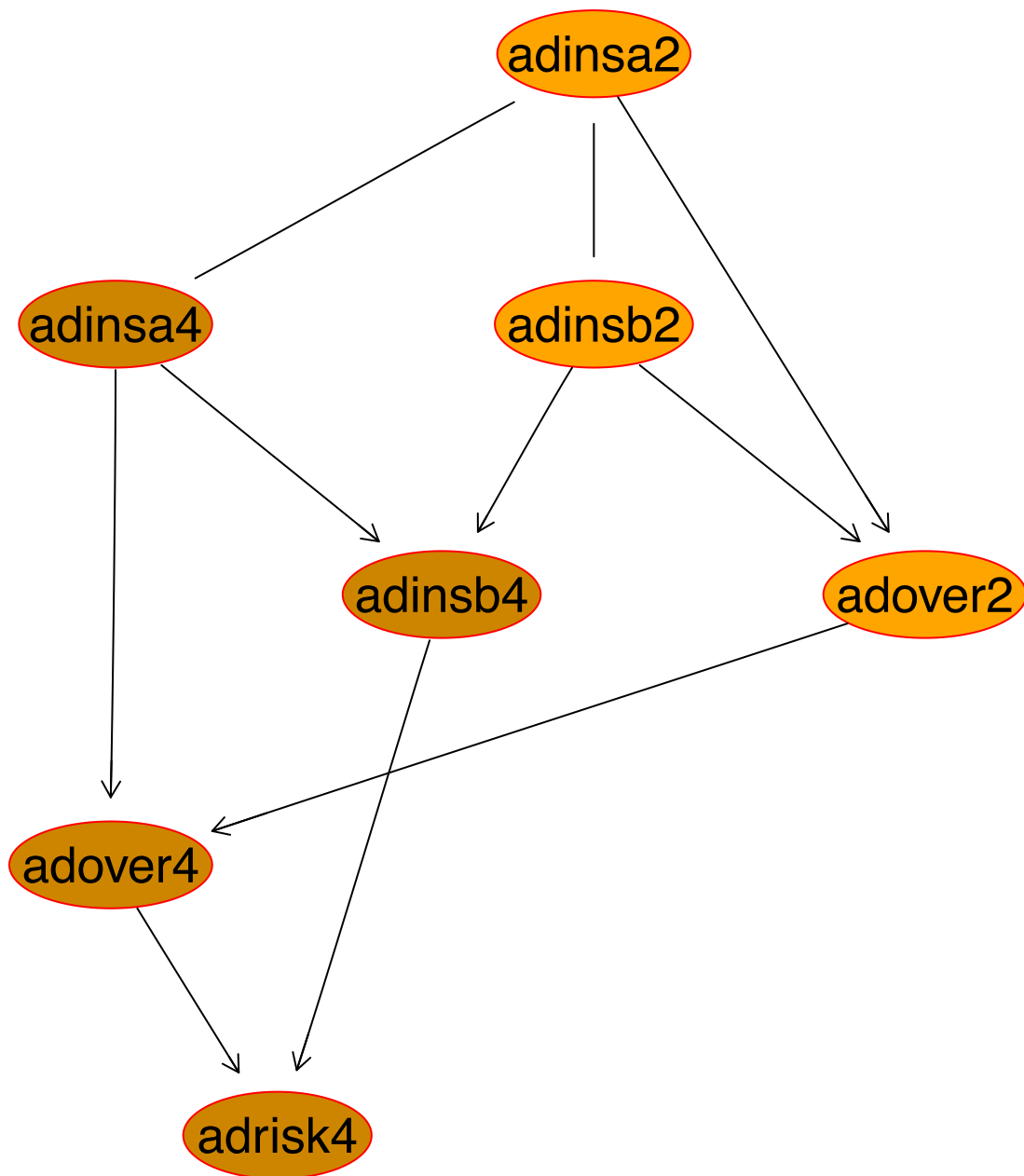

addprs2

adintr2

phq22

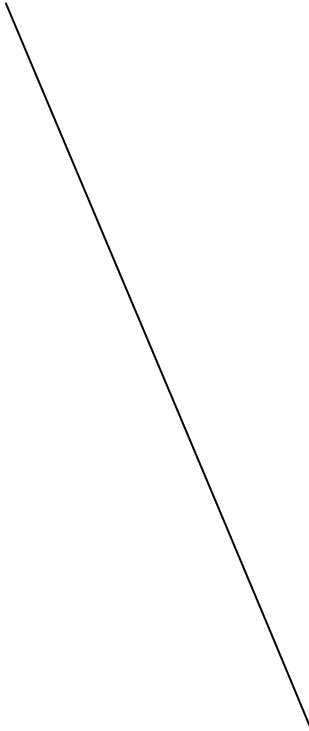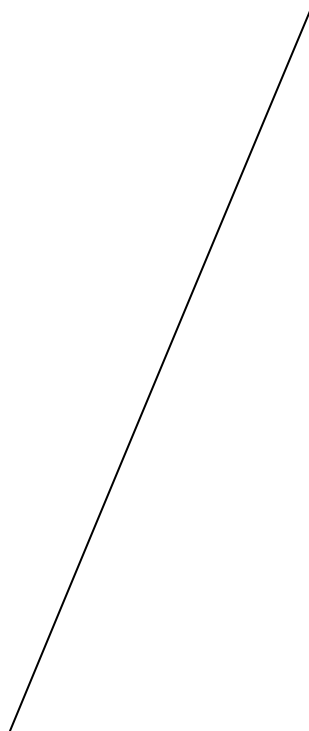

adexpl2

adhecr2

adlist2

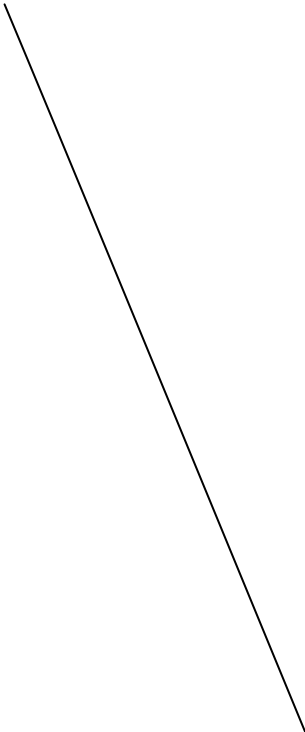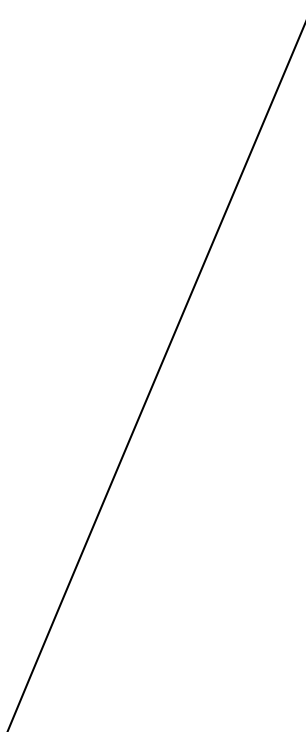

addprs4

adintr4

phq24

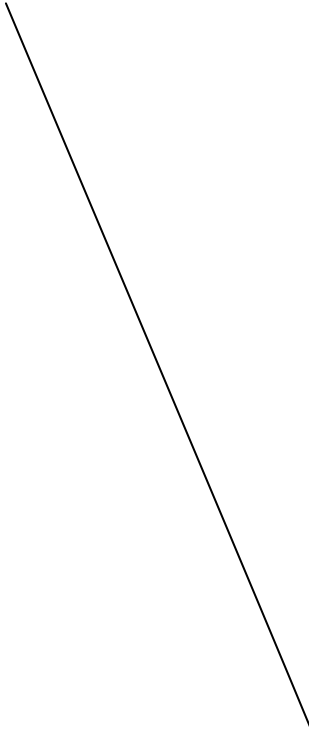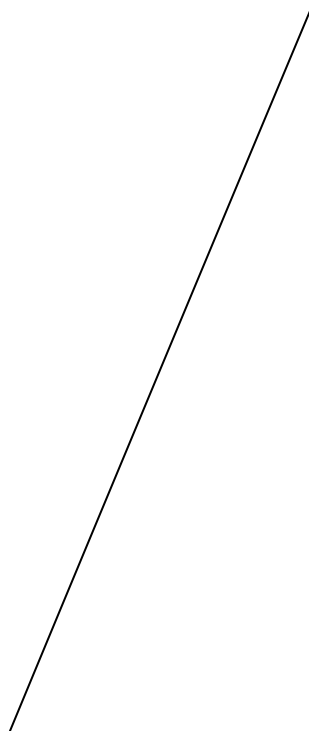

adnsmk2

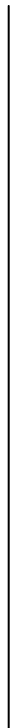

adsmok2

adnsmk4

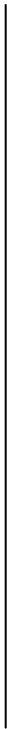

adsmok4

Supplement: Supplementary file 4 — Appendix 4. Networks of the measures of patient experiences and health status with the short names of the Medical Expenditure Panel Survey variables in the nodes. The results of the Bayesian network modeling with all connected networks. The original variables are labelled in the nodes. Corresponding variable names can be found in Additional file 1: Appendix 1. (PDF 72 kb) [file 12913_2017_2496_MOESM4_ESM.pdf]
