# Supplementary material for: A network perspective on patient experiences and health status: the Medical Expenditure Panel Survey 2004 to 2011
Source: BMC Health Serv Res. 2017 Aug 22;17:579. doi: 10.1186/s12913-017-2496-5 (PMC5567925; doi:10.1186/s12913-017-2496-5)
Supplement: Supplementary file 5 — Appendix 5. Networks of the measures of patient experiences and health status with the short names of the Medical Expenditure Panel Survey variables in the nodes. The results of the Bayesian network modeling with all connected networks. The names of the variables are labelled in the nodes. Corresponding variable names can be found in Additional file 1: Appendix 1. (PDF 76 kb) [file 12913_2017_2496_MOESM5_ESM.pdf]

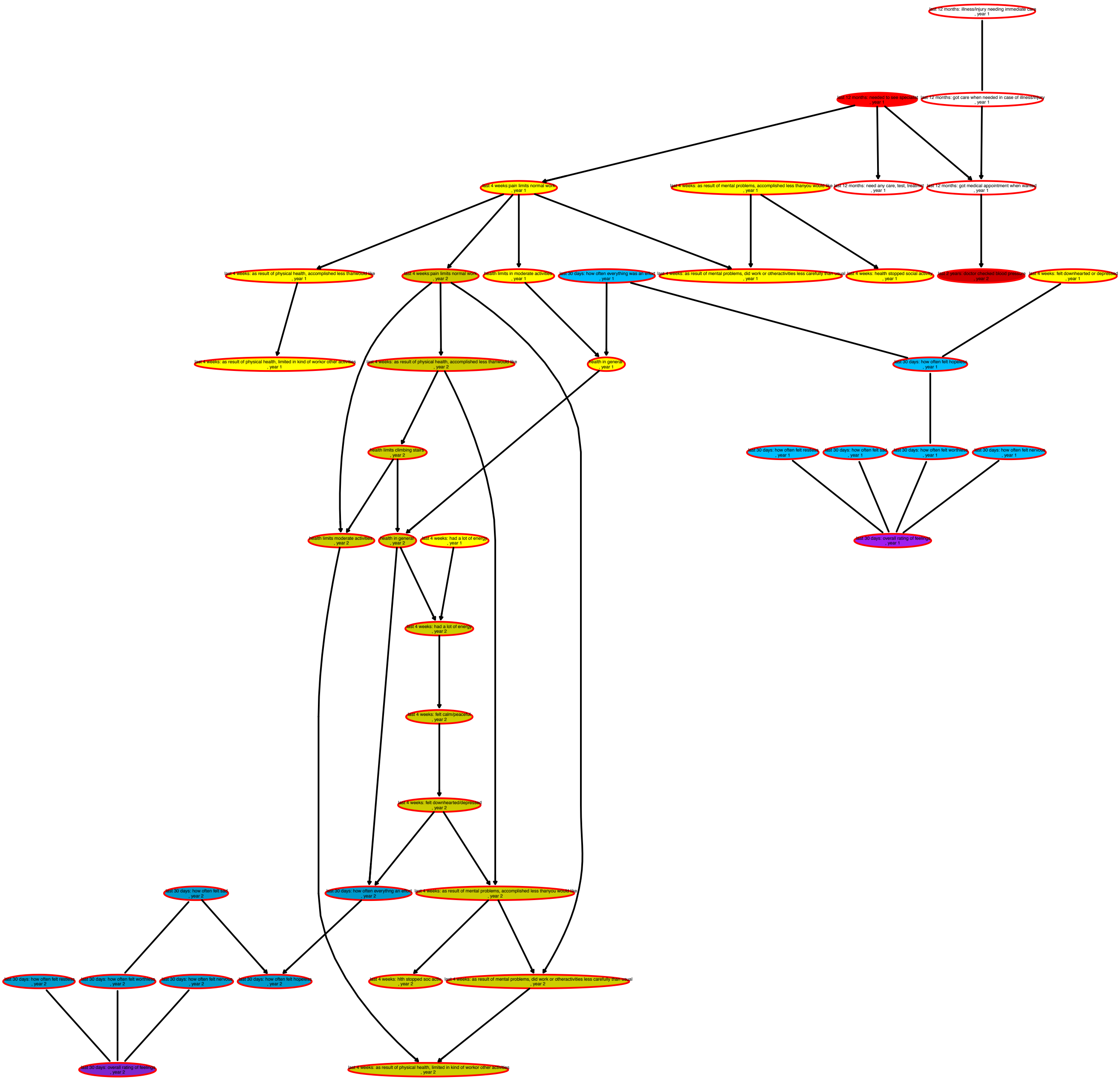

last 12 months: illness/injury needing immediate care , year 1
last 12 months: needed to see specialist , year 1
last 12 months: got care when needed in case of illness/injury , year 1
last 4 weeks:pain limits normal work , year 1
last 4 weeks: as result of mental problems, accomplished less thanyou would like , year 1
last 12 months: need any care, test, treatmnt , year 1
last 12 months: got medical appointment when wanted , year 1
last 4 weeks: as result of physical health, accomplished less thanwould like , year 1
last 4 weeks:pain limits normal work , year 2
health limits in moderate activities , year 1
last 30 days: how often everything was an effort , year 1
last 4 weeks: as result of mental problems, did work or otheractivities less carefully than usual , year 1
last 4 weeks: health stopped social activity , year 1
last 2 years: doctor checked blood pressure , year 2
last 4 weeks: felt downhearted or depressed , year 1
last 4 weeks: as result of physical health, limited in kind of workor other activities , year 1
last 4 weeks: as result of physical health, accomplished less thanwould like , year 2
health in general , year 1
last 30 days: how often felt hopeless , year 1
health limits climbing stairs , year 2
last 30 days: how often felt restless , year 1
last 30 days: how often felt sad , year 1
last 30 days: how often felt worthless , year 1
last 30 days: how often felt nervous , year 1
health limits moderate activities , year 2
health in general , year 2
last 4 weeks: had a lot of energy , year 1
last 30 days: overall rating of feelings , year 1
last 4 weeks: had a lot of energy , year 2
last 4 weeks: felt calm/peaceful , year 2
last 4 weeks: felt downhearted/depressed , year 2
last 30 days: how often felt sad , year 2
last 30 days: how often everythng an effort , year 2
last 4 weeks: as result of mental problems, accomplished less thanyou would like , year 2
last 30 days: how often felt restless , year 2
last 30 days: how often felt worthless , year 2
last 30 days: how often felt nervous , year 2
last 30 days: how often felt hopeless , year 2
last 4 weeks: hlth stopped soc activ , year 2
last 4 weeks: as result of mental problems, did work or otheractivities less carefully than usual , year 2
last 30 days: overall rating of feelings , year 2
last 4 weeks: as result of physical health, limited in kind of workor other activities , year 2

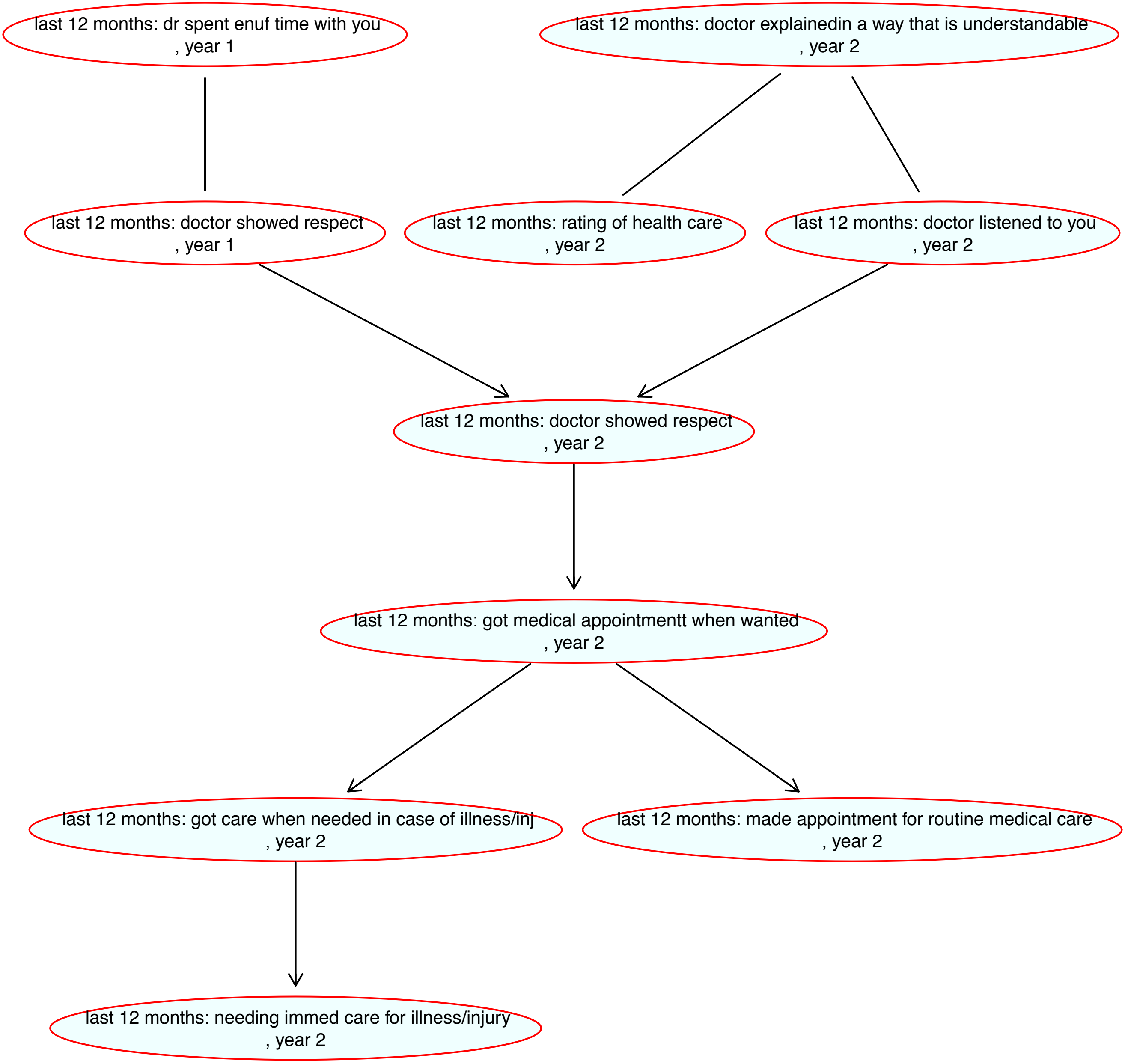
last 12 months: dr spent enuf time with you
, year 1
last 12 months: doctor explainedin a way that is understandable
, year 2
last 12 months: doctor showed respect
, year 1
last 12 months: rating of health care
, year 2
last 12 months: doctor listened to you
, year 2
last 12 months: doctor showed respect
, year 2
last 12 months: got medical appointmentt when wanted
, year 2
last 12 months: got care when needed in case of illness/inj
, year 2
last 12 months: made appointment for routine medical care
, year 2
last 12 months: needing immed care for illness/injury
, year 2

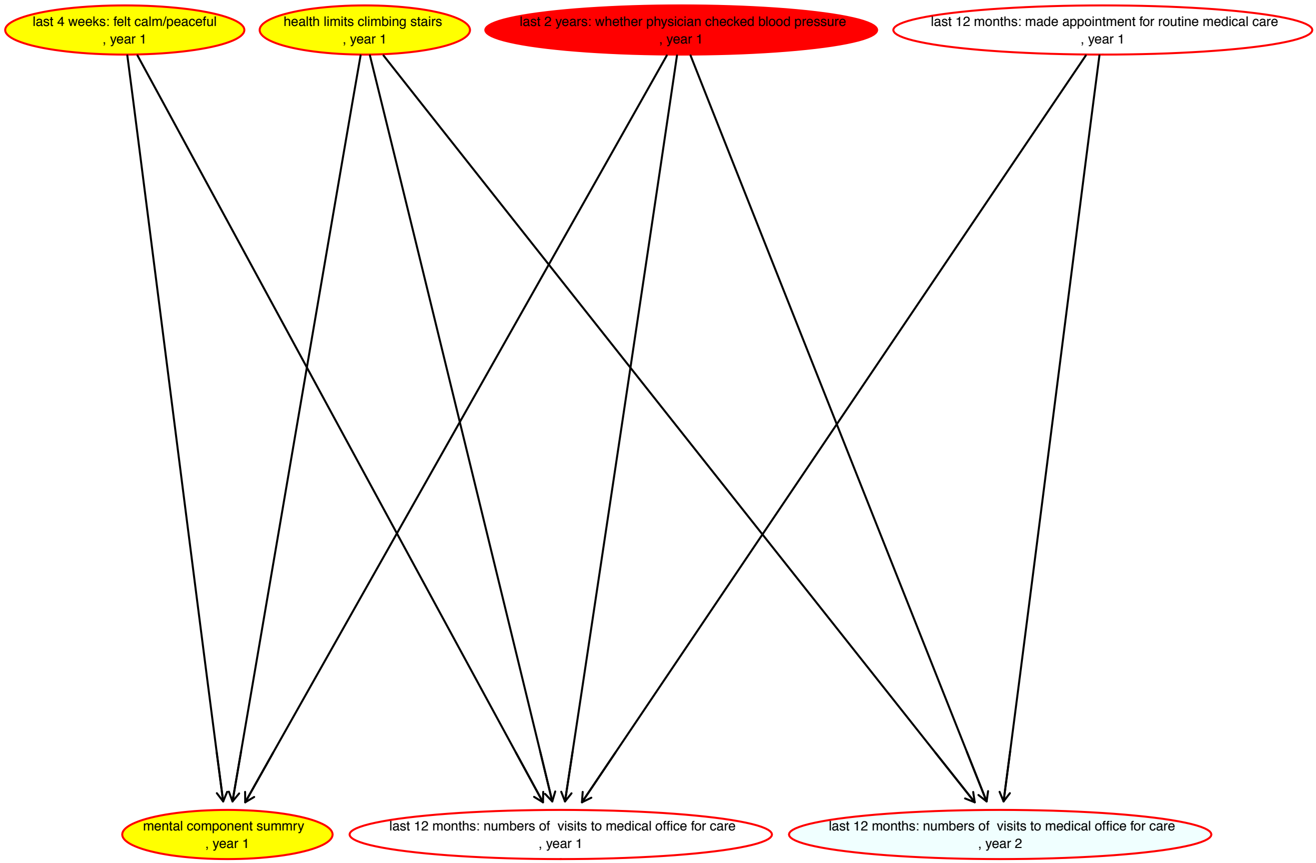
last 4 weeks: felt calm/peaceful
, year 1
health limits climbing stairs
, year 1
last 2 years: whether physician checked blood pressure
, year 1
last 12 months: made appointment for routine medical care
, year 1
mental component summry
, year 1
last 12 months: numbers of visits to medical office for care
, year 1
last 12 months: numbers of visits to medical office for care
, year 2

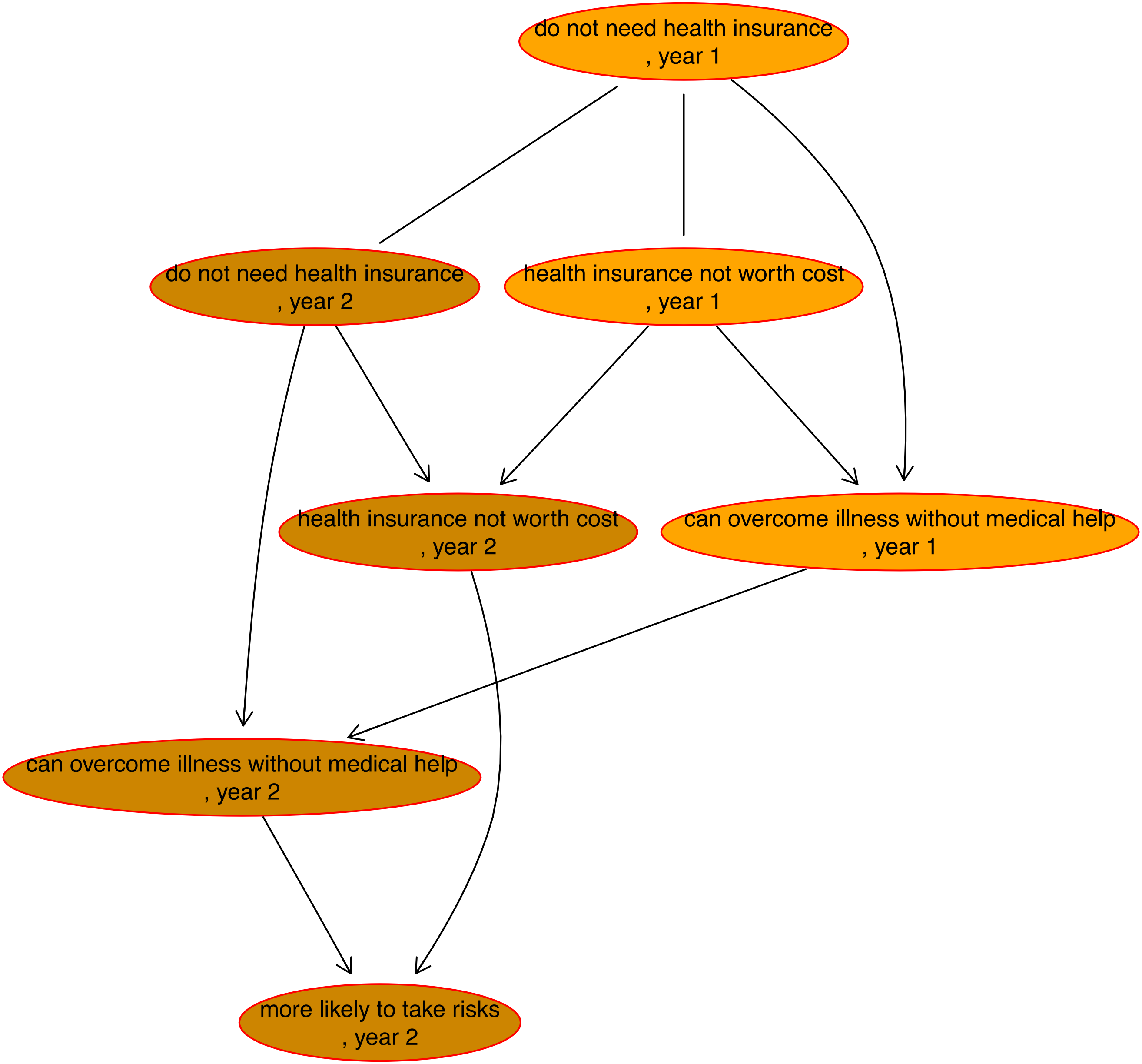
do not need health insurance
, year 1
do not need health insurance
, year 2
health insurance not worth cost
, year 1
health insurance not worth cost
, year 2
can overcome illness without medical help
, year 1
can overcome illness without medical help
, year 2
more likely to take risks
, year 2

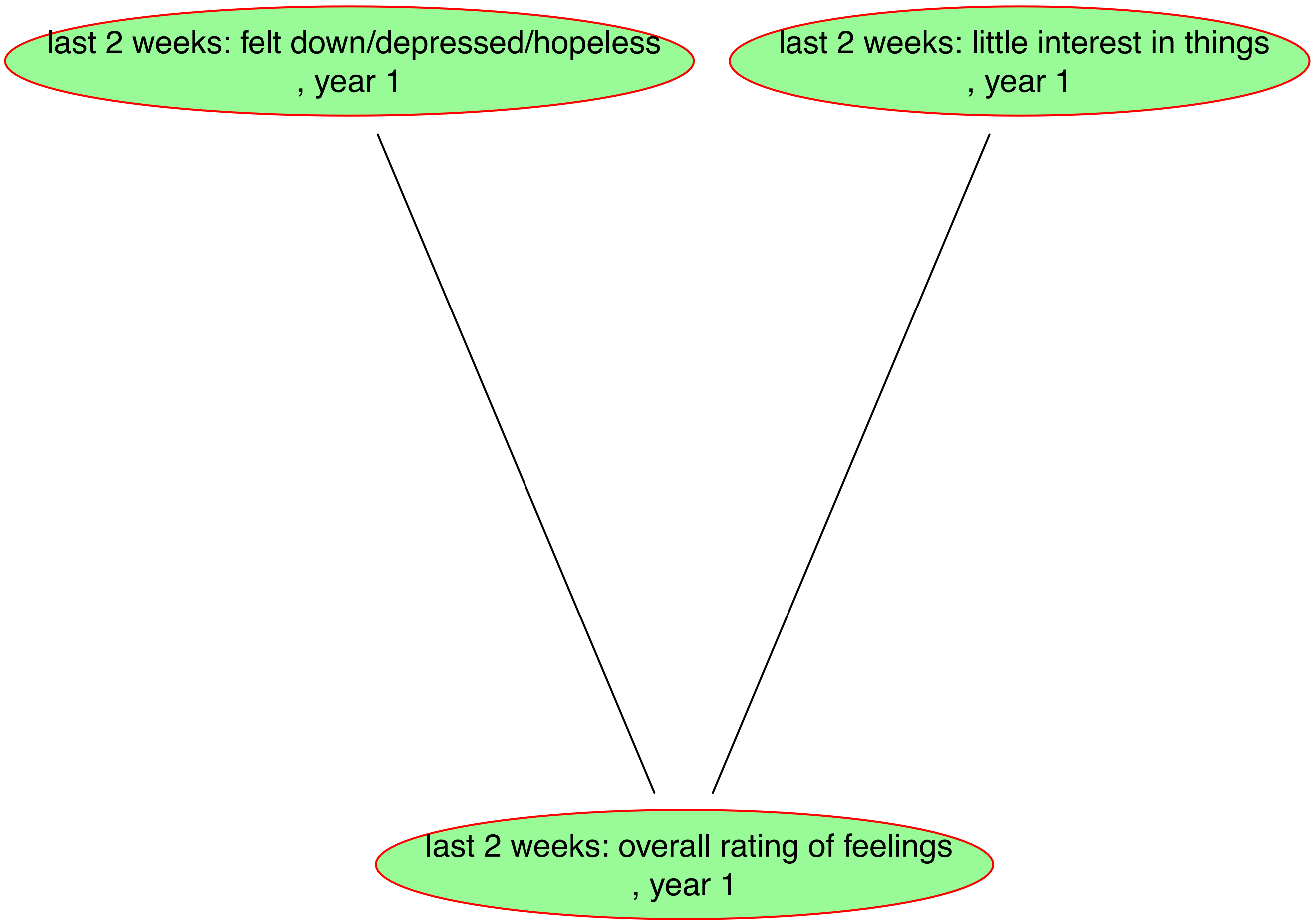
last 2 weeks: felt down/depressed/hopeless
, year 1
last 2 weeks: little interest in things
, year 1
last 2 weeks: overall rating of feelings
, year 1

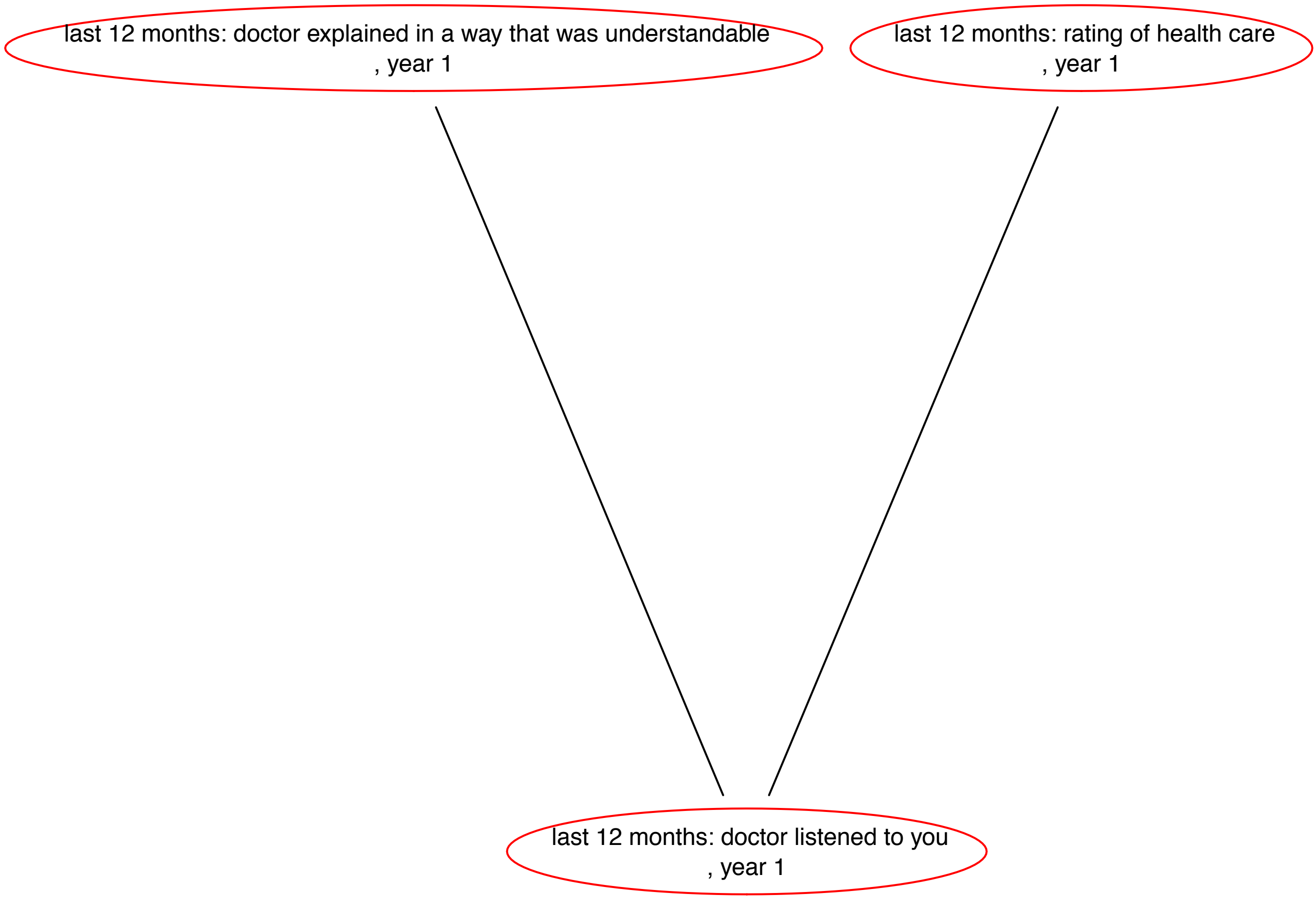
last 12 months: doctor explained in a way that was understandable
, year 1
last 12 months: rating of health care
, year 1
last 12 months: doctor listened to you
, year 1

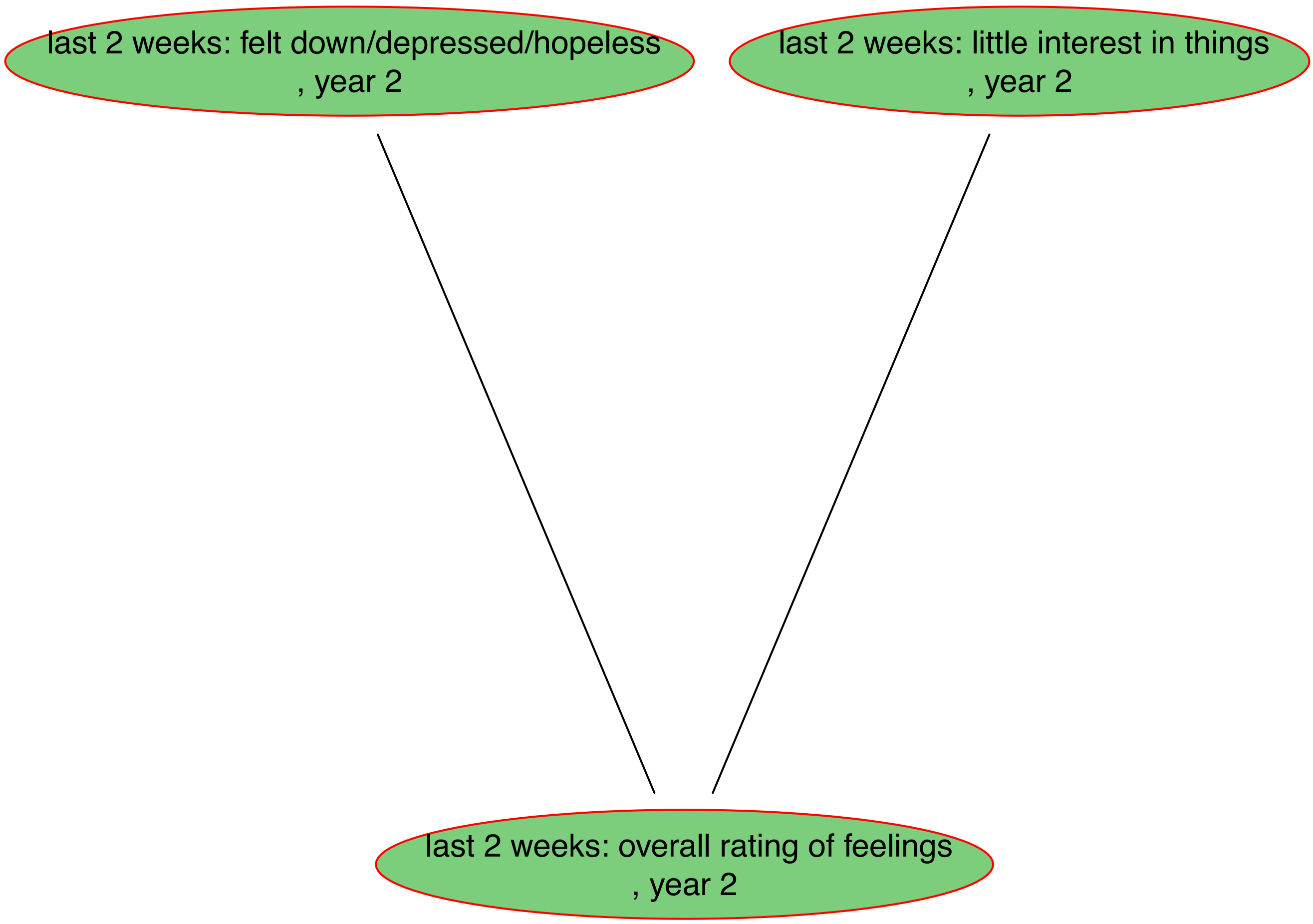
last 2 weeks: felt down/depressed/hopeless
, year 2
last 2 weeks: little interest in things
, year 2
last 2 weeks: overall rating of feelings
, year 2

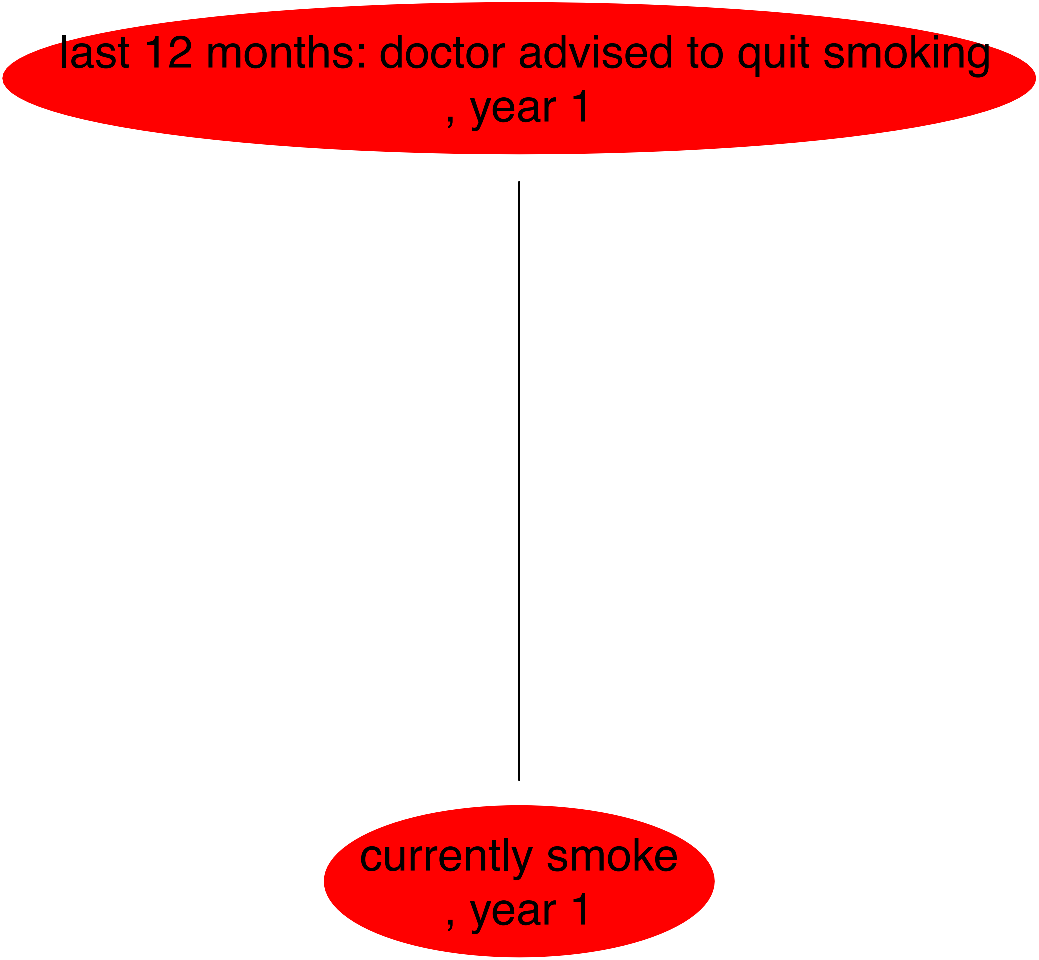
last 12 months: doctor advised to quit smoking
, year 1
currently smoke
, year 1

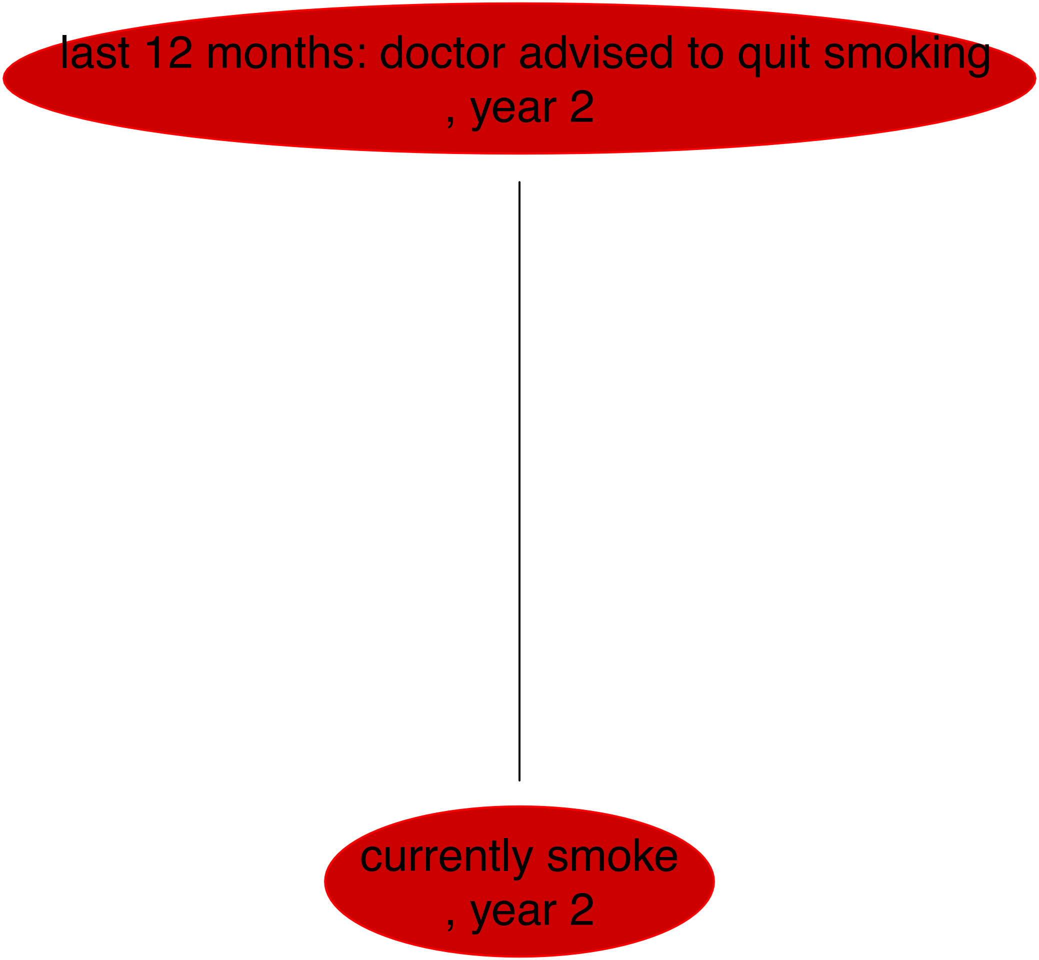
last 12 months: doctor advised to quit smoking
, year 2
currently smoke
, year 2
